# Regarding the first impact factor of the Journal of Applied Oral Science in the 2009 Journal Citation Reports Science Edition

**DOI:** 10.1590/S1678-77572010000300001

**Published:** 2010

**Authors:** Carlos F. Santos

**Affiliations:** 1 DDS, MSc, PhD, Associate Professor Editor-in-Chief Journal of Applied Oral Science.

Dear authors, reviewers and readers,

This editorial aims to acknowledge all of those who have contributed to our success with the Journal of Applied Oral Science (JAOS).

It is important to emphasize that the JAOS was the first journal from Latin America to be included in the Dentistry, Oral Surgery & Medicine category of the 2009 Journal Citation Reports (JCR) Science Edition. The JAOS has recently received its first impact factor of 0.384, being ranked 62nd out of the 64 journals listed in this prestigious index. We are aware of the greater responsibility we will face after our inclusion in the JCR. Nevertheless, we should have a natural improvement in the JAOS's impact factor due to its increased exposure to the entire scientific community. The more our journal is discovered the greater the probability is that our journal’s articles will be cited by authors throughout the entire scientific community, and thus, we can achieve a greater positive impact worldwide.

Our recent inclusions in MEDLINE/PubMed and ISI Web of Knowledge were instrumental in reaching this achievement. It is essential to remind our scientific community that this success is also a result of our indexation in the Scientific Electronic Library Online (SciELO), which provides free access to the full content of all the articles published. The SciELO network is a partnership among the State of São Paulo Research Foundation (FAPESP), the Latin America and Caribbean Center on Health Sciences Information (BIREME), and also includes many editors and national and international institutions related to scientific communication. This network must be continuously highlighted as an effective instrument in exposing and internationalizing many journals indexed in this database.

As we did during previous opportunities, we wish to thank all of those who supported the JAOS and provided us with invaluable contributions either as authors or reviewers. I also want to take this opportunity to express my gratitude to Professors Luiz Fernando Pegoraro and José Carlos Pereira, respectively former and current Dean of the Bauru School of Dentistry, for their continued support. I wish to also thank the National Council for Scientific and Technological Development (CNPq), the Foundation for the Coordination of Higher education and Graduate Training (CAPES) and the University of São Paulo for their financial support, which is crucial for the survival of our journal since we proudly belong to a non-profitable public institution without ties to commercial advertisers. Additionally, it is important to recognize the superb contributions from our colleagues in the area of Speech Language Pathology and Audiology.

Finally, I will always appreciate the brave team of employees at the Bauru School of Dentistry, who support and assist me on a daily basis managing the JAOS: the librarians Valéria Cristina Trindade Ferraz, José Roberto Plácido Amadei and Deborah Schmidt Capella Junqueira, the secretary Sônia Cláudia Pirola Rodrigues, the journalist Neimar Vitor Pavarini, and the cover designer Rubens Kazuo Kato. I hope some day I can retribute their devotion to the JAOS, the Bauru School of Dentistry and the University of São Paulo.

Please do not hesitate to contact me at editorjaos@fob.usp.br  with any criticisms, opinions or suggestions.

With my best regards,

**Carlos F. Santos**

DDS, MSc, PhD, Associate Professor

Editor-in-Chief

Journal of Applied Oral Science

